# Latency of breast cancer stigma during survivorship and its influencing factors: A qualitative study

**DOI:** 10.3389/fonc.2023.1075298

**Published:** 2023-03-14

**Authors:** Samar J. Melhem, Shereen Nabhani-Gebara, Reem Kayyali

**Affiliations:** School of Life Sciences, Pharmacy and Chemistry, Kingston University London, Kingston upon Thames, United Kingdom

**Keywords:** breast cancer, stigma, survivor, manifestations, Arab, Middle East, Jordan, factors (individual factors contextual factors)

## Abstract

**Introduction:**

Breast cancer diagnosis and treatment have been shown in studies to have a negative impact on patients’ physical, psychological, and social well-being, as well as overall quality of life. Psychologically, it’s linked to sadness, anxiety, and demoralisation. Stigma contributes to the hidden burden of breast cancer as a chronic illness. Research on the elements that breast cancer survivors encounter as influences on stigma associated to the disease is lacking. Based on the lived experiences of breast cancer survivors, this study sought to investigate the factors that lead to the manifestations of both self- and public breast cancer stigma.

**Methods:**

Individual semi-structured interviews with 24 patients diagnosed with breast cancer were performed, followed by five focus groups with 25 patients diagnosed with breast cancer. Interviews were verbatim transcribed and analysed using thematic framework analysis.

**Results:**

Two major themes have emerged from the data: a) Breast cancer stigma among breast cancer survivors, highlighting the various manifestations of stigma and the variables that influence them; including disease-related factors, patients’ views of cancer, public perceptions of breast cancer, family and interpersonal dynamics, and b) Stigma resilience and empowerment, emphasising the necessity of sociocultural transformation and coping strategies to preserve resilience.

**Conclusions:**

To improve the well-being of breast cancer survivors, practitioners and health policymakers should be aware of the breast cancer stigma that underpins patients’ emotional and behavioural outlooks and its potential consequences on patients’ quality of life. They need to develop interventions to address the different stages of cancer stigma taking into consideration sociocultural influences, norms, and beliefs.

## Introduction

Breast cancer is the most common female cancer worldwide and particularly in the Middle East (1). Breast cancer is Jordanian women’s most prevalent malignancy and the leading cause of cancer-related death (2). According to Jordan’s Cancer Registry (JCR), breast cancer is the most common cancer among females 38.5%, accounting for around 20.8% of all cancer diagnoses (2). Breast cancer in Jordan, similar to other low- and middle-income neighbouring countries, has a variety of distinguishing characteristics. The median age at presentation is 52 years, ten years younger than the median age in developed countries. Additionally, about a third of patients present with locally advanced or metastatic disease, highlighting the critical nature of early detection programmes (3).

Breast cancer diagnosis and treatment have been proven in studies to have a significant detrimental impact on patients’ physical, psychological, and social well-being, as well as and overall quality of life (4). Psychologically, it’s connected to depression, anxiety, and demoralisation in cancer patients (5). Breast cancer treatment modalities typically include a combination of surgery, chemotherapy, radiotherapy, targeted or hormonal therapy (6). Nearly every breast cancer patient has a surgical resection as part of their cancer treatment, which may cause disfigurement or changes in body image. Patients are stigmatised regardless of whether they have mastectomy or breast conserving surgery (4, 7). Throughout the course of the disease, the psychosocial burden of the disease and its ramifications such as poor social support, role functioning issues, and family crisis may further contribute to stigmatisation (8). As a result of recent breakthroughs in cancer treatment modalities and quality of care, the life-changing burden of cancer has shifted from treatment and mortality rates to a spectrum of medical and non-medical issues known as survivorship (9). Cancer survivors are confronted with a plethora of cancer treatment’s long-term adverse effects that may be a culprit in breast cancer stigmatisation. The most prevalent symptoms of which include fatigue, vasomotor symptoms, sexual dysfunction, musculoskeletal symptoms, neuropathy, and cognitive changes. Further, they may potentially develop cancer-induced conditions like osteoporosis, cardiac toxicity, obesity, infertility, and secondary malignancies (10). Breast cancer management as a chronic disease necessitates a focus on stigma as a contributor to the illness’s hidden burden (11). Stigma is a term that refers to a sense of isolation, exclusion, devaluation, and criticism that occurs throughout a social process or personal experience and has an impact on the results of physical, psychological, and social adjustment (4). Disease-related stigma is “the stigmatisation of an illness, which may be directed towards an individual or a group of people with the illness.” (5) It has detrimental consequences from diagnosis to completion (12), and to some extent, it will deter patients from seeking medical help, causing them to frequently attempt to conceal their condition from others and delay seeking medical care (13). Internalisation of cancer-related stigma results in low self-esteem and poor mental health, smaller social networks and less social potential to obtain support, and increased anticipation of social rejection, all of which negatively impact quality of life (5). Cancer is considered a socially stigmatised disease in many cultures, but some aspects of stigma are more prevalent than others (14). Previous research suggested a pervasive stigma associated with cancer. According to an early study, 52% of women with breast cancer in the United States of America reported feeling avoided or dreaded (14). Recent research has proven the high prevalence of cancer stigma in the U.S. and across many nations, including but not limited to, Japan, England, and Korea (15–18). Marlow and Wardle (19) used a web-based questionnaire to investigate public attitudes toward cancer patients in the United Kingdom. The researchers defined policy opposition (e.g., more government funding spent on cancer care and treatment) and financial discrimination as unfavourable reactions to cancer patients in addition to avoidance (e.g., It is acceptable for banks to refuse to make loans to people with cancer). Similarly, Balmer et al. (20) investigated how non-cancer adult populations in high-income nations react to cancer, concluding that individuals frequently avoid discussing it. Because cancer causes fear, healthy people rarely bring it up in ordinary conversation. Typically, cancer is rarely discussed until it becomes a personal concern (21). Existing studies mostly focus on prevention, access/barriers to medical care, and treatment experience, with few evaluating cancer stigmata during survivorship. As medical treatment has improved dramatically over the past few decades, a growing proportion of cancer patients are now survivors. More research on the long-term health effects of cancer stigma is required to fully understand what patients go through from cancer diagnosis through survivorship (14). Moreover, whereas previous literature focused on the disease itself as a stigma that affected all individuals equally, it is now more pertinent to analyse cancer-related characteristics such as cancer type, visibility, and the likelihood that the disease will impede the individual’s ability to achieve personal goals or interact in social contexts. A modern concept of cancer-related stigma should focus on cancer survivors as “targets,” with cognizance of how others see them, their interpretations social settings, and their motives and goals (22).

There is a dearth of research examining the most prevalent aspects and influencing factors of breast cancer-related stigma experienced by breast cancer survivors. In this study, we use qualitative methods to bridge this gap and offer new insights that can be used to empower health programme developers and policymakers by identifying crucial areas in which this population could benefit from education to change perceptions and dispel misinformation and by developing culturally relevant breast health promotion programmes for Jordan and comparable Middle Eastern Arabic cultures.

## Methods and materials

### Study design

The study used semi-structured interviews and focus groups to collect qualitative data as part of a larger project on breast cancer survivors’ experiences and a patient-centred holistic digital platform for cancer supportive care. The qualitative methodology was chosen to examine of cancer survivors’ lived experiences, attitudes, and behaviours (23). Semi-structured interviews were used to obtain women’s experiences and perspectives (24, 25). Focus groups were used to explore detailed attitudes and experiences of participants, revealing similarities and differences as perspectives and views were shared (26). The interview schedule was based on *a priori* framework and a review of studies examining experiences with breast cancer survivors. The topic guide was influenced by the Trajectory of Breast Cancer (TBC) framework developed by Simit et al. (27), who synthesised stories from a heterogeneous group of women with different types of breast cancer. They varied in age, disease stage, treatment regimen, country of origin, and other characteristics (27). This review’s scope allowed for a broad analysis of international breast cancer narratives. It provided a breast cancer timeline so researchers to consider breast cancer experiences relative to TBC time-points. Specifically, the framework included the time-point ‘survivorship,’ which is important given the rising number of breast cancer survivors (27). This highlights the complicated illness of breast cancer and the need for support before, during, and after active treatment (5). The topic guide covered three sections: the first section explored cancer challenges and the patient journey; the second section focused on communication with healthcare professionals, treatment challenges, and follow up; and the third section explored social support sources, care pathway constraints, coping strategies, and online resources for adjusting to the illness. The interview guide includes prompts. A pilot interview with a breast cancer survivor was audiotaped and transcribed verbatim before the study began to ensure clarity, flow, format, and structure of the questions. This interview was excluded from the final analysis. See the supplementary document for the full interview guide.

### Ethical considerations

This qualitative study was approved by Jordan University Hospital (JUH) approval number: (10/2019/8990) and Kingston’s university ethical requirements for scientific research (Approval number/1416). Before the study, participants gave written and verbal consent. Specifically, verbal consent was obtained from members participating in online group discussions.

### Data collection

Participants were recruited from oncology outpatient clinics at Jordan University Hospital, a large tertiary semi-governmental hospital in Amman. Purposeful sampling was used to reflect the whole population of breast cancer survivors by identifying those articulate, reflective, and willing to share their experience with the interviewer (28). Inclusion criteria were: 1) diagnosis of primary breast cancer with or without recurrence from all stages (I-IV), 2) adult female aged 18 and over, 3) completed curative or salvage therapy (i.e., in follow up stage or surveillance) and 4) cognitively able to comprehend the implications of consent and participate in an interview or focus group. Two breast oncologists identified 73 patients for the study. The cancer nurse specialist called them and invited them to participate. The nurse sent them a predesigned participant information sheet (PIS) on the study’s aims and objectives *via* WhatsApp. Nine patients declined to participate. The lead female investigator (SJM), who has considerable experience in qualitative research, contacted the 64 patients who joined to discuss the interviews and address any queries. Participants were also assured of the anonymity and confidentiality of all information collected for the study. Participants’ demographics and medical records were collected before interviews/focus groups. The investigator (SJM) assigned participants a 1:1 interview or a focus group. Thirty-three patients preferred an interview while 31 were scheduled for a focus group. Before conducting the interviews, 15 patients dropped out, leaving 24 for the semi-structured 1:1 interview, and 25 participated in five online focus groups. Except for participants 4 and 24, who participated in a focus group., all were interviewed once (29) The data coding continued until theoretical saturation was reached and no new concepts were discovered by repeated reviewing and coding. After codes were complete, themes were developed. Themes were saturated after 22 interviews and 4 focus groups. All scheduled interviews and focus groups were held as planned (30, 31). Face-to-face Interviews were conducted between (2/1/2020 and 28/2/2020) and lasted from 42-147 minutes (average time 67 minutes). During the interviews, no one else was present. Participants were informed of the research’s purpose and signed consent forms were collected before the interviews. Focus groups were held online between (30/4/2020 and 18/6/2020) *via* Skype due to COVID-19 restrictions. For focus groups, verbal consent was obtained from all members before starting. Flexible question order and probing questions were used to explore some issues in depth.

### Data analysis

All interviews were audiotaped in Jordanian Arabic, transcribed verbatim by the first author (SJM) and later translated to English (SJM). Two bilingual colleagues (RK and SN-G) who are fluent in Arabic and English reviewed the translated transcripts. Field notes were only taken after a 1:1 interview. No transcripts were returned to participants for feedback who were unknown to the interviewer. The study used framework methodology, a form of qualitative thematic analysis that integrates five interconnected steps to provide a systematic and rigorous audit route (32–34). The framework approach to data analysis entails five stages: familiarisation with all data, establishment of a thematic framework (based on *a priori* objectives, concerns, and topics expressed by participants), indexing (systematic coding of the data), charting (grouping the data thematically in charts and comparing within and across participants), and mapping and interpretation (exploration of the themes in relation to overarching patterns and explanation of the findings) (32). The framework methodology is compatible with deductive and inductive reasoning, and Gale et al. (32) advocate a combined approach. Deductive approaches use pre-selected themes and codes based on prior literature, pre-existing beliefs, or the research question, while inductive approaches derive themes from data through open (unrestricted) coding and theme refinement. According to Gale et al. (32), a mixed strategy is optimal when the research attempts to discover unanticipated aspects of the participants’ experience or how they assign meaning to phenomena while exploring some specific challenges. This paper uses a hybrid and will focus on two overarching themes related to breast cancer stigmatisation during survivorship, as revealed by the 1:1 interview and focus groups. The “health stigma and discrimination framework” was used as a theoretical framework to analyse emerging data on breast cancer stigma (5). To establish initial themes, one author (SJM) coded all transcripts. Two authors (RK and SN-G) contributed to the conceptualization and development of the thematic framework and consistency review of emerging themes and codes. Regular researcher meetings ensured rigour of preliminary codes. Overarching themes are organised into specific themes and subthemes based on data analysis (35, 36). To provide “referential adequacy” and “tell the story” and show data analysis conclusions, quotations were used throughout the text. This reveals the researchers’ understanding, differentiates the research participants’ voices from the researchers’, and allows readers to build their own interpretations (35, 37). All quotes are cited as interview/FG (survivor number, age in years). To evaluate, apply, and synthesise study results, the Standards for Reporting Qualitative Research SRQR was used (38).

## Results

### Participants

In total, 49 women with median age at diagnosis was 46 (range, 32 to 65) participated in interviews or focus groups. Twenty-nine were married, 11 were single, 7 were divorced/separated, and 2 were widowed. Most participants were diagnosed in stages I or II, and the length of survivorship ranged from 1 to 23 years. Most of the study population was highly educated with (12/49) having a master’s or doctorate degree and (24/49) holding an undergraduate degree. The characteristics of the participants are listed in **Tables 1**, **2**.

**Table 1:** Breast cancer survivors’ interviews (n=24).

ID	Age at Diagnosis (Years)	Age (years)	Education‡	Marital Status	No. of children	Occupation	Family history/Gene positivity	Cancer Stage	Treatment modality◊	Trajectory of care	Years of survivorship
**1**	**44**	**51**	**undergraduate**	**married**	**3**	**nurse**	**no**	**II**	**S^R^+C+R+H**	**follow up**	**7**
**2**	**37**	**49**	**undergraduate**	**single**	**0**	**office administrator**	**yes**	**I**	**S^c^+C+R+H**	**follow up**	**12**
**3**	**51**	**58**	**undergraduate**	**married**	**3**	**housewife**	**no**	**II**	**S^R^+C+T**	**follow up**	**7**
4**	**49**	**55**	**undergraduate**	**married**	**3**	**self-employed**	**no**	**II**	**S^R^+C+R+T**	**follow up**	**6**
**5**	**43**	**48**	**masters**	**separated**	**2**	**housewife**	**yes**	**II**	**S^R^+C+R+T**	**follow up**	**5**
**6**	**40**	**45**	**secondary**	**married**	**1**	**housewife**	**unknown**	**II**	**S^R^+C+R+T**	**follow up**	**5**
**7**	**46**	**52**	**undergraduate**	**married**	**2**	**teacher**	**yes**	**II**	**S^R^+C+R+H**	**follow up**	**6**
**8**	**43**	**44**	**secondary**	**married**	**3**	**housewife**	**unknown**	**II**	**S^R^+C**	**Follow up**	**1**
**9**	**39**	**43**	**undergraduate**	**separated**	**2**	**teacher**	**yes**	**II**	**S^R^+C+R+H**	**follow up**	**4**
**10**	**43**	**45**	**undergraduate**	**single**	**0**	**government employee**	**yes**	**0**	**S^c^+R+H**	**follow up**	**2**
**11**	**40**	**43**	**undergraduate**	**married**	**3**	**teacher**	**yes**	**II**	**S^R^ +C+R+H**	**follow up**	**3**
**12**	**47**	**52**	**secondary**	**married**	**6**	**housewife**	**no**	**II**	**S^R^ +C+R+H**	**follow up**	**5**
**13**	**51**	**54**	**undergraduate**	**married**	**3**	**teacher**	**yes**	**II**	**S^R^+C+R+H+T**	**follow up**	**3**
**14**	**55**	**67**	**secondary**	**married**	**7**	**retired/teacher**	**unknown**	**III**	**S^c^+C^neo^+R+T**	**follow up**	**12**
**15**	**49**	**55**	**masters**	**separated**	**1**	**pharmacist**	**no**	**II**	**S^c^+C+R+H**	**follow up**	**6**
**16**	**49**	**57**	**doctorate**	**married**	**4**	**academia (linguistics)**	**yes**	**II**	**S^w^+C^neo^+R+H**	**follow up**	**8**
**17**	**36**	**38**	**undergraduate**	**single**	**0**	**temporary unemployed**	**no/genetic mutation**	**III Triple negative**	**S^R^+C**	**follow up**	**2**
**18**	**60**	**73**	**general education**	**married**	**4**	**housewife**	**unknown**	**II**	**S^R^+C+R+H**	**follow up**	**13**
**19**	**42**	**45**	**undergraduate**	**married**	**2**	**unemployed (Tourism)**	**yes**	**II**	**S^c^+C+R+H**	**follow up**	**3**
**20**	**60**	**71**	**undergraduate**	**single**	**0**	**nutritionist**	**unknown**	**II**	**S^c^+C+R+H**	**follow up**	**11**
**21**	**40**	**49**	**undergraduate**	**married**	**5**	**housewife**	**no**	**IV**	**S^R^+C+R+H+2n line chemo**	**follow up**	**9**
**22**	**32**	**37**	**undergraduate**	**single**	**0**	**non-governmental organisation (NGO)**	**no/genetic mutation**	**I**	**Bilateral mastectomy+C+R+H**	**follow up**	**5**
**23**	**43**	**46**	**undergraduate**	**separated**	**2**	**civil engineer**	**no**	**I**	**S^R^+C+R+H**	**follow up**	**3**
24*	**35**	**37**	**doctorate**	**divorced**	**0**	**psychologist/social worker**	**no**	**I**	**S^c^+C+H**	**follow up**	**2**

‡ (39) https://www.scholaro.com/pro/Countries/Jordan/Education-System.

◊ SR, Modified Radical Mastectomy.

Sw, wide local excision.

Sc, Breast conserving surgery.

R, Radiotherapy.

C, Chemotherapy.

Cneo, Neoadjuvant chemotherapy.

H, Hormonal therapy.

T, Targeted therapy.

**Table 2:** Breast cancer survivors’ focus groups (n=25).

ID	Age at Diagnosis (Years)	Age (years)	Education‡	Marital Status	No. of children	Occupation	Family history/Gene positivity	Cancer Stage	Treatment modality◊	Trajectory of care	Years of survivorship
**FG1_S1**	**42**	**44**	**doctorate**	**single**	**0**	**nurse/academic**	**yes/genetic mutation**	**II**	**S^c^+C+R+H**	**under treatment (aesthetics)**	**2**
FG1_S2*	**35**	**37**	**doctorate**	**divorced**	**0**	**psychologist/social worker**	**no**	**I**	**S^c^+C+R+H**	**follow up**	**2**
**FG1_S3**	**52**	**58**	**secondary**	**married**	**3**	**housewife**	**no**	**III**	**C^neo^+S+T**	**follow up**	**6**
**FG1_S4**	**41**	**46**	**masters**	**married**	**4**	**math teacher**	**yes**	**I**	**S^c^ +C+R**	**follow up**	**5**
**FG2_S1**	**44**	**52**	**masters**	**married**	**3**	**government employee (civil engineer)**	**yes**	**I**	**S^c^+C+H**	**follow up**	**8**
**FG2_S2**	**57**	**68**	**general education**	**married**	**7**	**housewife**	**no**	**I**	**S^w^+C**	**follow up**	**11**
**FG2_S3**	**50**	**54**	**undergraduate**	**married**	**2**	**retired (lab technician)**	**no**	**II**	**S^R^+C+R+T**	**follow up**	**4**
**FG2_S4**	**49**	**55**	**undergraduate**	**married**	**3**	**self-employed**	**no**	**II**	**S^R^+C+R+T**	**follow up**	**6**
**FG3_S1**	**50**	**56**	**undergraduate**	**married**	**3**	**housewife**	**no**	**II**	**S^R^+C+H**	**follow up**	**6**
**FG3_S2**	**32**	**39**	**undergraduate**	**divorced**	**0**	**software engineer**	**yes**	**II**	**S^R^+C+R+H**	**Follow up**	**7**
**FG3_S3**	**51**	**54**	**secondary**	**Married**	**5**	**housewife**	**No**	**III**	**S+C^neo^+R+T**	**Follow up**	**3**
**FG3_S4**	**58**	**64**	**general education**	**widowed**	**0**	**cooking from home**	**No**	**II**	**S^R^+C+R+H+T**	**Follow up**	**6**
**FG3_S5**	**62**	**67**	**secondary**	**widowed**	**4**	**housewife**	**No**	**II**	**S^R^+C+R+T**	**Follow up**	**5**
**FG3_S6**	**48**	**57**	**masters**	**single**	**0**	**nutritionist**	**No**	**II**	**S^w^+C+R+H+T**	**Follow up**	**9**
**FG4_S1**	**52**	**59**	**masters**	**single**	**0**	**retired nurse**	**Yes**	**II**	**S^R^+C+R+H**	**Follow up**	**7**
**FG4_S2**	**60**	**73**	**secondary**	**married**	**0**	**housewife**	**No**	**II**	**S^R^+C+H**	**Follow up**	**13**
**FG4_S3**	**38**	**42**	**undergraduate**	**married**	**1**	**pharmacist**	**No**	**I**	**S^c^+C+R+H**	**Follow up**	**4**
**FG4_S4**	**65**	**69**	**secondary**	**married**	**2**	**housewife**	**No**	**III**	**S^R^+C+R+H+T**	**Follow up**	**4**
**FG4_S5**	**38**	**41**	**undergraduate**	**married**	**0**	**human resources**	**Yes**	**II**	**S^R^+C+R+H**	**Follow up**	**3**
**FG5_S1**	**35**	**38**	**secondary**	**single**	**0**	**administrative clerk**	**No**	**II**	**S^R^+C+R+H**	**Follow up**	**3**
**FG5_S2**	**48**	**71**	**undergraduate**	**married**	**5**	**retired (social worker)**	**No**	**I**	**S^R^+C+R+H**	**Follow up**	**23**
**FG5_S3**	**51**	**56**	**undergraduate**	**married**	**4**	**retired/Government (legal affairs)**	**No**	**II**	**S^R^+C+R+H**	**Follow up**	**5**
**FG5_S4**	**48**	**58**	**masters**	**married**	**2**	**retired/banker**	**No**	**I**	**S^c^+R+H**	**Follow up**	**10**
**FG5_S5**	**42**	**45**	**undergraduate**	**single**	**0**	**accounting auditor**	**No**	**II**	**S^c^+C+R+H**	**Follow up**	**3**
**FG5_S6**	**41**	**43**	**masters**	**single**	**0**	**computer engineer**	**Yes**	**I**	**S^c^+R+H**	**Follow up**	**2**

‡ (39) https://www.scholaro.com/pro/Countries/Jordan/Education-System.

◊ SR, Modified Radical Mastectomy.

Sw, wide local excision.

Sc, Breast conserving surgery.

R, Radiotherapy.

C, Chemotherapy.

Cneo, Neoadjuvant chemotherapy.

H, Hormonal therapy.

T, Targeted therapy.

### Themes

**Figure 1:**
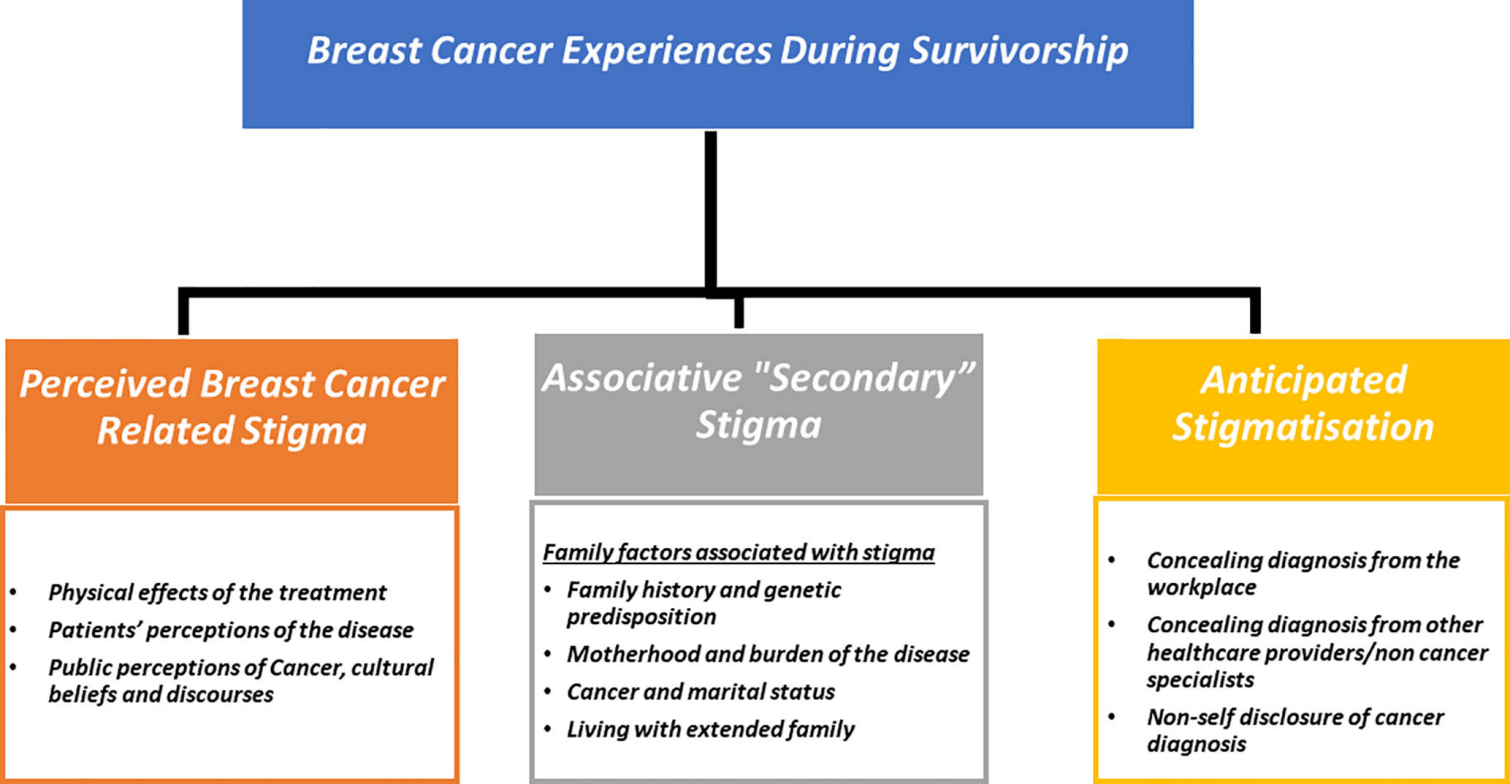
Breast cancer stigma during survivorship.

**Figure 2:**
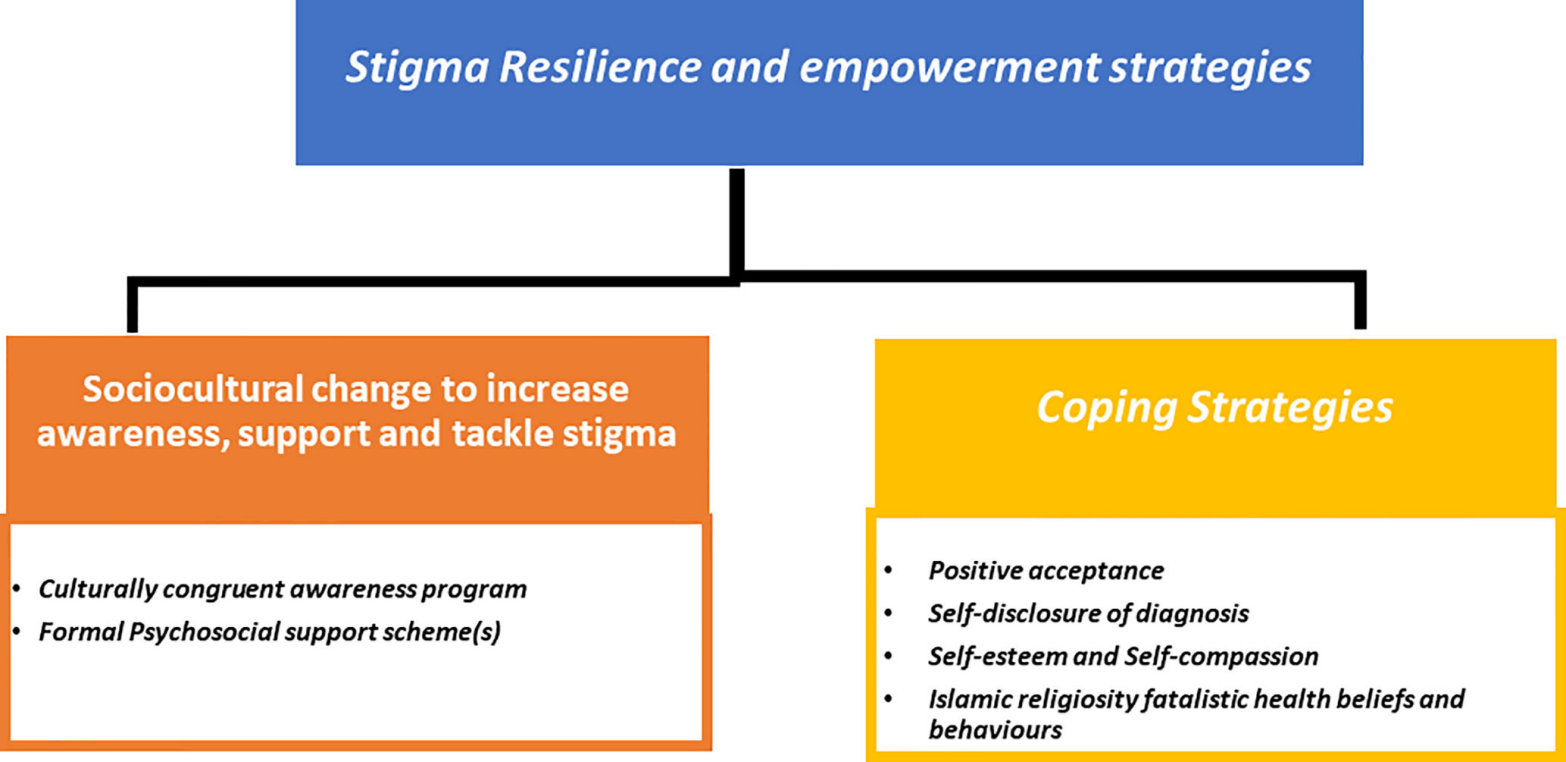
Stigma resilience and empowerment strategies.

Two overarching themes emerged from the data 1) Breast Cancer Stigma experiences during survivorship (**Figure 1**); 2) Stigma resilience and empowerment mechanisms (**Figure 2**).

#### Theme 1: Breast cancer stigma experiences during survivorship

The stigmatisation process during survivorship involves a complex interaction of disease-related issues, patients’ views of the illness, sociocultural factors, interpersonal and family relationships, all of which contribute to the stigma of breast cancer as a “lived reality.” Three subthemes were identified in relation to breast cancer-related stigma manifestations throughout survivorship: a) perceived cancer - related stigma (PCS) b) secondary stigma or associative stigma; and c) anticipated stigmatisation. The quotations in **Boxes 1**-**4** provide further evidence for these themes.

##### Perceived cancer-related stigma (PCS)

The subtheme, perceived breast cancer-related stigma during survivorship, referred to how cancer survivors’ perceptions (attitudes, beliefs, and experiences) are impacted by a variety of influential factors and circumstances that contribute to a stigma latency during survivorship. These issues include the physical effects of cancer treatment (visible short-term effects of treatment-induced physical changes such as alopecia, nail changes, skin changes, and post-surgical sequelae), long term effects of treatment, and the failure or inadequacy of financial assistance for aesthetic and reconstructive surgery.

*“I lost my hair after the second cycle. I didn’t expect how swiftly it would fall out. Afterward, I cried so much, and I broke the mirror. My whole family, even my husband, came to my side to help me feel better. I remember that time; I’m glad it’s over. You might think that after 13 years, all of the negative effects will have gone away, but it will still leave scars in one’s heart.” Interview 1 (S1, 51)*

*“I took the mirror out of the bathroom because just looking at myself reminds me of my condition, it’s like a disease signature.”. FG1 (S5, 41)*

*“I’m sure someone will notice something wrong when they look at me. I used to be sensitive, but not any longer. This is a naive way of thinking; cancer problems extend much beyond body image. For example, my relationships with others have changed; I used to have many friends, but now I only have a few since they don’t understand what it’s like to live with this condition for a long period”. Interview 7 (S7, 52)*

Other quotes related to the physical effects of the treatment are provided in box 1.1. Several participants reported experiencing lymphedema, osteoporosis, a lack of strength, and pain as the most common long-term physical side effects of the treatment. They believe that these side effects are inevitable and that they have little control over them, which leaves them feeling helpless.

*“Since I’ve been sick for nine years, I’m feeling weak and tired. My bones hurt, my arm hurts, my sight isn’t what it used to be, and every part of my body hurts. I can’t tell anyone because they’ll be bored, and they won’t get me. The doctors won’t be able to fix everything even if I come to the hospital. Some people say that this condition can be treated, but the effects can’t be treated.” Interview 21 (S21, 49)*

Younger survivors in reproductive age (24/49 survivors, ≤ 45 years at the time of diagnosis) indicated that the long-term effects of treatment, particularly the adverse effects of hormone medication, robbed them of their feeling of youth and femininity. They gained weight and felt and looked older than their actual age.

*“My period stopped four years ago after I finished my therapy. The doctors informed me it could be because of the therapy, which has made me frustrated; I no longer feel like a young lady.” FG 5 (S6, 43)*

Several participants expressed that, despite their eligibility for breast reconstruction or having a prosthesis, they could not afford it, in contrast to the costs of therapy, which are covered by the Ministry of Health. In addition, prothesis failure and its complications exacerbated the issue and hindered the patients’ ability to maintain or improve their body image.

*“I just wished the doctor would show me a picture of how my breast will look after mastectomy or a video; I don’t like how it looks, but I have to accept it. My doctor has told me that reconstruction is an option, but that surgery is out of the question for me. When I asked, they told me that the Ministry of Health only covers treatment costs, and that reconstruction is not a treatment.” Interview 8 (S8, 44)*

*“Having a silicon implant to boost my self-esteem and confidence didn’t work for two years, so now I’m going to the clinic to get rid of the problems that came from it.” Interview 19 (S19, 45)*

Additionally, patients’ perceptions of the illness, which include (cancer identity and alienation; different experiences and emotions associated with cancer; cancer as a vicious cycle and religious fatalism), contributed to perceived cancer-related stigma.

As a group, cancer survivors share a common experience because of their body changes. Some participants claimed that having cancer gives them a new identity, which has been associated with social detachment or alienation, such as not participating in sad ceremonies or funerals, avoiding people and not actively interacting with peer patients in an effort to avoid adopting this identity.

*“I don’t like going to the cancer centre because you just see cancer patients; it depresses me; that’s why I chose to be treated here in the hospital, because there are other patients like diabetics and arthritis, not just cancer.” FG1 (S1, 44)*

According to cancer survivors, the disease was associated with cancer-specific feelings and emotions (fear, distress, anxiety, sadness, and despair). These views originate from treatment, incapacitation, recurrence, and death, and might be reinforcing elements in the disease’s perceived stigmatisation.

*“When I hear of someone who lost their battle with cancer or who had a recurrence, it reminds me of myself and my journey, and how difficult it was, …………, I still feel sad for this sad ending.” Interview 16 (S16, 57)*

*“Yes, as you progress, you’ll feel better and your morale will rise, especially when they say you’re cured and everyone is happy after finishing the treatment stage, yes, the big fears and worries go away, but there is still an entrenched fear of cancer returning, which is why I come here for follow-ups every three months Interview 23 (S23, 46)*

*“I don’t feel down all the time, but I become really worried between follow-ups, especially if there are new results, or if I have certain symptoms, such as adverse effects, or if I am waiting for an imaging report, waiting for test results is stressful and overwhelming” Interview 4 (S4, 55)*

Other informants viewed cancer as an empty circle that will come back.

*“The doctor told me it was triple negative, but I didn’t understand what that meant at the time, they don’t tell you everything, just drop by drop, I came here to the clinic because I finished chemotherapy 3 months ago and they ran a PET scan, the doctors discovered a likely spread in the pelvis, I don’t know what to say, I don’t want to stay at home because my family is even more upset than me, especially my sister who knew everything from the beginning, it’s an empty circle.” Interview 17 (S17, 38)*

Additional quotes related to patients’ perceptions of the disease are provided in **Box 1**.

According to the participants, societal perceptions of cancer and cultural ideas and discourses such as the ingrained nature of cancer fear, exhibiting pity as an inferiority signal instead of compassion, and society’s prejudice and judgmental unfavourable stance on cancer all play a role in internalising cancer stigma.

*“I didn’t tell anyone about my condition because I didn’t want to have that look of pity in their eyes that truly hurts, that they feel sorry for me.” FG 5 (S4, 58)*

Further, some cancer survivors’ outlook on the disease was influenced by their religious beliefs. Fatalism is the belief that one’s health is beyond one’s ability to control. For instance, cancer fatalism is the belief that death is inevitable when cancer is present. In a religious setting, fatalism also refers to the belief that health outcomes are predetermined or under the control of a higher power, primarily God.

*“ I don’t know why I got the disease, and I believe in the prophet Mohammad hadith.” What is meant for us will find its way to us. What is not meant for us will never come to us. It is Allah’s will, and this is my fate, I think that it’s my destiny to get this disease and I can’t do anything about it”. Interview 21 (S21, 49)*

*“My sisters don’t care and don’t want to listen to me when I told them about screening, they said that no one knows the future except God, they said they might have other kinds of cancer, there are many causes and one death.” Interview 13 (S13, 54)*

Additional quotes on community’s perceptions of the disease and cultural and religious beliefs influencing perceptions are provided in **Box 1**.

**BOX 1 Perceived cancer-related stigma (PCS).**

Physical effects of the treatment

1.1. “When my doctor told me that even if he performed reconstruction surgery, my breasts would never be the same as before, I burst into tears, but I reminded myself that this was the best we could do. He told me that you are fortunate because many women are not candidates for reconstructive surgery. So, I'd never be the same as I used to be. The illness alters your personality. Not just one thing, but several.” Interview 8 (S8, 44)

1.2. “I was beautiful and athletic before cancer, but I can't seem to get back into shape. As a result of Tamoxifen, I gained a lot of weight and experienced hot flashes; my doctor advised me to continue taking it for at least 7 years or the cancer would return. It's giving me a lot of side effects, but I have to take it because my fear of cancer returning is so strong that if I don't have to go through chemotherapy again, you better kill me, and I'm feeling helpless.” Interview 19 (S19, 45)

Patients’ perceptions of the disease

1.3. "Seeing another cancer patient having chemotherapy or looking like a cancer patient will remind you of yourself and your experience; other people will not know or comprehend how the sickness transforms us from the inside out. Interview 4 (S4, 55)

1.4. “In the beginning, everyone told me that this was a dangerous disease, and I needed to undergo regular check-ups... " I used to feel a lump in my breast when I put my hand there. Of course, I was very afraid, and my nerves were very tired. I was on edge at the time." Interview 12 (S12, 52)

1.5. “My greatest concern is that the disease would recur, not because I am terrified of death, but because I do not want to start over with treatments, particularly chemotherapy, which is excruciating and dreadful." Interview 14 (S14, 67)

1.6. “Cancer, in general, makes you fragile and emotional; it also changes your perspective on life; you begin to see things differently; we keep it to ourselves. FG4 (S4, 69)

Society’s perception of the disease and cultural beliefs and discourses

1.7. " On Facebook, people only discuss cancer after someone dies from it; in our culture, they only mention two points: someone had cancer and, after a while, she lost her struggle with cancer; they don't even use the word cancer." FG1 (S2, 37)

1.8."I know they're not intentionally trying to make me feel bad, but I can no longer go to the funerals of my family members who have died. I don't know how I became this way; before my cancer diagnosis, I was a very gregarious person, but I can't stand by and see others grieve or go to funerals. People who are close to me won't understand, and social norms and duties make me feel like I'm a prisoner of this illness. I often wait 3 or 4 days before attending sorrowful events since my heart can't handle it". FG4 (S1, 59)

1.9. “I know God will reward me for all the suffering. I start to think about it and it makes me feel bad. I know that our lives are in God's hands.”. Interview 16 (S16, 57)

##### Secondary or associative stigma

The second subtheme, “associative stigma” as a manifestation of breast cancer stigmatisation, relates to the experience of stigma by cancer survivors’ family members. According to the responses, associative stigma may be influenced by family history and genetic predisposition, motherhood and disease burden, cancer, and marital status, and living with extended family.

*“When I received my diagnosis, it was the worst shock of my life; I was just 42 years old and a university doctor, and I love my job. My siblings were really supportive, and they went out of their way to help me. But there is a hidden side of the story; as I already said, I am BRACA2 positive, and my aunt died from the same condition. I’m still not sure how it will affect us socially, but as for me, I’m keeping it to myself, only few people know, and as a family, we’re keeping it to ourselves.” FG1 (S1, 44)*

*“In general, people are more aware that breast cancer can spread in families. This isn’t true for everyone, but only those who have been properly educated on the subject. In the end, this could affect someone’s chances of getting married or her social status, because society isn’t willing to change, and the collective mind can’t be changed. It’s because if they know that you have a gene, they might think that you’re a “flawed person.” FG1 (S2, 37)*

*“I was divorced after 11 years of marriage and without children; someone wants to propose to me, and I want to be a mother; I underwent oocytes cryopreservation since the doctor told me it was the only way to get pregnant and have children in the future. However, there is a slim probability that it will be successful. I’m not sure if he’d still come back and propose. You are also aware of the changes that occur following surgery. It’s difficult to put into words.” Interview 24 (S24, 37)*

Further quotes on associative stigma can be found in **Box 2**.

**BOX 2 Associative “secondary stigma”.**

***2.1.* “**I don't know who is going to take care of my autistic child, I am the only one who can take care of him, crying.” Interview 8 (S8, 44)

**2.2**. “When I got the disease, my little child was 2 years old and my daughter was 7,now my daughter is 17 and can take care of her brother if I’m gone, I’m grateful to God and my doctor that I was able to survive despite all the suffering, I told my doctor I didn’t want to get chemo again.so he prescribed a mouth pill, we decided not to tell anyone it had returned, and my children have no idea what it means. I don't want them to hear about the condition from other people because they are still young children.” Interview 21 (S21, 49)

2.3. "I don’t talk much about it with my family as long as I am ok. We only share positive thoughts, no one like to talk about something related to cancer, I am concerned about my daughters, I don’t want them to experience what happened to me, it’s a brutal disease. Interview 13 (S13, 54)

2.4. “My ex-husband was quite supportive during therapy; now, after four years, he has married another; I simply informed him that I wish to divorce”. Interview 5 (S5. 48)

2.5. "I am divorced, my family is upset because we see marriage as a long-term commitment, no one in our family is divorced, I have no choice, and I am unable to begin a new relationship." FG3 (S2, 39)

2.6. “I was diagnosed when I was 32 years old; I am still alive, but I have lost many things; my mother and brother are aware of my disease; my father has another wife, and we are a large family; they are unaware……. “I had both of my breasts removed. Because everyone in our small town knows everyone else, many of whom are family, we didn't tell anyone; I'm not sure if they did a genetic test, but my doctor advised me to get both breasts removed. I didn't have the energy to inquire why at the time.” Interview 22 (S22, 37)

##### Anticipated stigmatisation

The third subtheme explores the elements that contribute to the expression of anticipated stigmatisation among cancer survivors. These elements contribute to cancer diagnosis concealment in a variety of contexts as an adaptive response to identity threat or as a strategy for stigma resilience. Several survivors stated that they concealed their cancer diagnosis at work or from non-cancer healthcare providers. Additionally, several women acknowledged concealing their diagnosis from extended family members and disclosing it selectively to others.

*“I had annual mammograms for seven years because I am educated, and I used to go with my friends. After noticing a discharge from my nipple, I rushed to the hospital, where I was referred to an oncologist. He told me to contact him if the discharge changed colour, such as if it turned bloody. When the discharge turned brown, I was terrified. Following a biopsy and an MRI, my doctor discovered ductal carcinoma in situ and performed ductal excision. Seven years were squandered performing mammograms. And they could only identify it with an MRI; I told my doctor I would have paid a thousand JD (1400$) to perform the test myself, he said it is not routinely required. I cried a lot after the doctor removed all of my breast tissue and removed my nipple. I asked my doctor to never use the term (cancer) in the medical report, simply the phrase “mammary ducts excision,” as I do not want anyone in the workplace to know I had cancer, I want to get promoted and pursue my career.” Interview 10 (S10, 45)*

*“During chemotherapy, I used to cover my face when I visit the cancer centre since my students are training there and I don’t want them to notice me, now I only come to see the surgeon here in the clinic” FG1 (S1, 44)*

*“I work in the private sector; when I informed them, I wanted to start my treatment procedure, they refused to give me an unpaid vacation; they stated I could come back and apply after I recovered.” Unfortunately, they hired someone else because, as you know, women in our community aren’t supposed to support their families financially, but I don’t want to lose my job. Then I applied for another Job, not telling them I had cancer because it was none of their business.” FG5 (S6, 43)*

*“I can’t work as long as I used to, I don’t have the stamina, I can’t tell my supervisor what’s going on with me, like hot flashes, how my hormones and period changes are impacting me, and all my co-workers are guys.” Interview 23 (S23, 46)*

*“I didn’t feel this way because I never told anyone about my disease. I also didn’t quit my job, so I’m always working.” Interview 10 (S10, 45)*

*“I never told a dentist or other doctor that I had cancer. I didn’t even tell the pharmacist when I bought a drug.” Interview 10 (S10, 45)*

*“My husband told me not to tell anyone from his family because they might start interfering and putting pressures, my parents are old, so I only told them that it’s a benign lump because my sister had breast cancer and died 15 years ago.” Interview 19 (S19, 45)*

Further quotes to support the sub-theme anticipated stigmatisation are provided in **Box 3**.

**BOX 3 Anticipated stigmatisation.**

3.1. "I took annual vacation and didn't tell anyone at work except my manager, who was quite encouraging and I told her to keep it a secret." Interview 7 (S7, 52)

3.2." When I was diagnosed I started planning my life for the next 5 years and I told my sons, I thought it's hopeless and I decided to retire early since the treatment takes a long time and I don't want to be in this working environment, and I didn't tell anybody". FG5 (S4,58)

3.3."I just didn't tell anyone except a few close friends and family members because I knew they would talk about it behind my back and hurt us. I can't take any more emotional pain because I'm already full.” Interview 16 (S16, 57)

#### Theme 2: Stigma resilience and empowerment

Despite the wide stigma experienced and perceived, the second theme describes the strategies and adaptations used by some cancer survivors to overcome stigmatisation adversities (**Figure 2**). This theme includes a sub-theme on coping strategies, which covers the following: a) positive acceptance; “*Cancer has changed me profoundly, and I became a better person as a result. Because of what I’ve been through, I’ll be more attentive to the needs of others, and I’ll always do my best to get others close to me in order for them to remember how beautiful I was, because life is short and there should be something influential, we can do for others, because we’re only here for a little time.” Interview 9 (S9,43) b*) self-disclosure of diagnosis; “*I was diagnosed with cancer and treated at the hospital where I work. Everyone knows and has been very supportive, especially my doctor. This has given me the chance to switch to a more positive role. My doctor always calls me to support women who come to the clinic. When they see me, they cheer up and say, “Impossible! You never look as you had it before!” Interview 2 (S 2,49)* c) self-esteem and self-compassion; “ *You hear a lot of stories, some of which make you sad. I tell myself how lucky I am compared to those who have advanced cases or diseases that can’t be cured……… even though I’m suffering, where I’ve been and how strong I was when I was smashed to pieces.”* Another informant said, “*I tell myself, life is beautiful,” all the time. Sometimes I can’t feel it, but I keep reminding myself of my blessings. If nothing is good, there is bad and there is worse. This keeps me going.*” *Interview 15 (S15,55)* and d) Islamic religiosity’s fatalistic health views and behaviour- According to some participants, this is a response to cancer as a chronic illness that brings them calm and tranquilly and aids in coping with disease adversities and social challenges.

*“As I already said, the tumour was discovered in a miraculous manner. (She said, “I went to the hospital for an abdominal CT scan because I was experiencing kidney colic pain. The hospital called me two days later to tell me that they had found a lump in the lower part of my breast. That’s how my story began.) When I think of how early the disease was discovered, I am reminded of God’s mercy and care, there are some things in life that we can’t control or predict, and we will be tested in different ways in this life and rewarded in the afterlife.” Interview 24 (S24, 37)*

More quotes on this sub-theme are provided in **Box 4**.

The other subtheme is related to sociocultural change to increase awareness, support and tackle stigma. Cancer survivors indicated that empowerment and increased engagement in support programmes necessitate a sociocultural transformation at the national level, as well as the development of formal psychosocial support services.

*“No one cares about those who survive. They only get treatment…. which is good compared to other countries, but we don’t have formal psychosocial programmes to help empower women and meet their needs.” FG 3(S2,39)*

*“We need new ways to speak about cancer in the media that reflect the reality of the disease and the challenges in our society and to motivate people to react positively and become more engaged; also, many women in our country are disempowered and require support, as well as novel ways to reach out to the public.” FG5 (S2, 71)*

Other quotes to support this sub-theme are provided in **Box 4**.

**BOX 4 Stigma resilience and empowerment.**

Coping strategies

Islamic religiosity’s fatalistic health views and behaviour

4.1. “I always remind myself how much God cares about me.”, Interview 15 (S15, 55)

4.2 “Because our ages and our lives are all in God’s hands, we should be faithful, which is very vital, I mean acceptance and faith, will be half the way to mental relief and wellbeing. Though the diagnosis itself is a major milestone, life goes on and we must cope.” FG 5 (S2, 71)

4.3.”I see this struggle as a blessing in disguise because it brought me closer to God. He is the only one who knows the pain, and he will pay me back in the afterlife.” Interview 2 (S2, 49)

4.4. “I like to be around people, and I don’t care what they think of me because I become more self-conscious. Since my diagnosis, my life has been guided by a hadith from the Prophet Mohammed. (Never a believer is stricken with a discomfort, an illness, an anxiety, a grief or mental worry or even the pricking of a thorn but Allah will expiate his sins on account of his patience).” FG5 (S2, 71)

Sociocultural change to increase awareness, support and tackle stigma

4.5. “Before I had the disease, I used to think like every one, every one fears cancer, our society think that all cancers are the same, it leads to death eventually, every year we see this ultra-positive pink campaign “promise us you will get checked”, we hear lots of positivity like it will be highly curable, but when I get into the experience the society still fear the disease, they don’t know what does it mean to go through all this, they don’t know how our lives changed upside down, we have awareness campaigns but we need support programs and the awareness programs should focus on the rest of the journey not only screening and treatment.” FG5 (S4, 58)

4.6. “It’s going around like the flu! When I come to follow up, I see new women coming in to check for symptoms; nearly every one of us knows someone who has the disease; we have a screening programme, but I believe it is insufficient; I believe we need more effective awareness programmes to educate the public, family, and caregivers; and these initiatives should be tailored to our cultural and societal norms.” FG1 (S2, 37)

4.7.”We can’t make national support programmes or support groups until we fix cultural problems. If we don’t, many women won’t join…”FG1 (S1, 44)

## Discussion

This study addressed the social construction of breast cancer-related stigma as a “lived reality” through Jordanian breast cancer survivors’ narratives and experiences. We sought to comprehend self and public breast cancer stigma, as well as the elements that contribute to its manifestations, which stem from cancer survivors’ conceptions, beliefs, and lived experiences with the disease as a chronic condition. Other factors include social interactions, cultural and religious beliefs, societal norms, and discourse. Two overarching themes emerged from the data. The first theme- breast cancer stigma during survivorship - portrayed the various manifestations of cancer-related stigma during survivorship as a complex interaction of disease-related factors, family dynamics and interpersonal relationships, patient perceptions of the disease, and public perceptions and sociocultural beliefs and attitudes. Our findings revealed that PCS (5) was influenced by short- and long-term cancer-related physical consequences (for example, alopecia, mastectomy, and weight gain). Previous research indicated that regaining physical and psychological health following a breast cancer diagnosis may be a long and challenging process for survivors (especially in terms of body image issues) (4). Financial constraints to restoring a healthy body image or the failure of cosmetic procedures, according to our results, are among the factors that contribute to disease stigmatisation. A substantial body of evidence suggests that PCS adversely impacts breast cancer patients’ attitudes, behaviours, psychological, and quality of life (4, 40, 41). Almost all the breast cancer patients who underwent treatment suffered from significant side effects. The majority of survivors experienced significant and long-lasting physical alterations, clinical symptoms, sleep disturbances, and lifestyle changes that reduced their quality of life (8). Therefore, survivors’ inability to adjust and manage long-term effects of treatment such as weight gain, fatigue, and osteoporosis also contribute to the inability to restore physical well-being. PCS is prevalent among women with breast cancer and stigmatisation includes negative feelings and attitudes (such as frustration, despair and depressive emotions, negative attitude and decreased healthcare seeking behaviour) as well as social avoidance of survivors (42). As the disease causes physical, psychological, and social morbidity, cancer stigma has been identified as an impediment to health promotion (43). The entrenched fear of the disease at the both the individual and public level, as well as the threat of death as it is perceived as a vicious cycle due to fear of recurrence or progression, may contribute to the stigma associated with cancer. This study corroborates with previous studies indicating that breast cancer and its treatment bestow a long-term identity change and confer a “cancer identity, “which is the basis for this judgement (11). Manifestations included social isolation, avoiding other cancer patients, and fatalistic beliefs and appearance-based judgments. The stigma associated with breast cancer is a concern. PCS has negative impacts on patients and their families at home, in the community, and at work. Anticipated stigmatisation refers to the anticipation of prejudice from others if a health problem becomes known (5). This is manifested by concealing diagnosis in different situations. This can be explained by the identity threat model proposed by Kapp et al. (44) and its components “situational threat” and “personal attributions” that enable cancer survivors to evaluate potentially harmful cues in a variety of situations (e.g., the workplace, social interactions/contexts, and the healthcare system) in different ways. Disease concealment may promote resilience and adaptation by allowing cancer survivors to selectively process disease origins, features, and effects. Although stigma sensitivity due to cancer-induced physical alterations is unique from other social stigmas, its psychosocial manifestations may be conducive to social stigma. Patients who are more sensitive or conscious of being stigmatised because of their cancer may feel more threatened by their identity. The desire to protect themselves from any threats that they perceive as endangering their professional or personal development contributes to the stigma experienced by cancer survivors, which may sustain their self-esteem in the short term but perpetuate negative stereotypes in the long run (22, 44). Cancer has a unique stigma, yet there is no universal agreement that having cancer places a person in a single stigmatised group or category. Cancer survivors vary in how much they internalise their cancer identity as part of their self-identification and this may be influenced by cultural and religious beliefs about cancer’s lethality, poor reputation, and taking control of one’s life. Those who internalise and identify with stigmatised beliefs, whether actual or perceived, may identify more as a cancer “sufferer” than as a cancer “survivor,” which conveys a positive connotation (44). The latency and dynamic nature of breast cancer-related stigma may be explained using Jones et al. (45) theory of social stigma that identified six distinguishing characteristics: concealability, course, disruptiveness, aesthetic quality, origin, and risk. “Concealability” relates to how easy patients may conceal their cancer. For example, breast cancer mastectomy is less concealable than gastrointestinal cancer. “Course” focuses on people’s perceptions about cancer prognosis and death. Cancer is commonly linked with death, even though advances in cancer therapy have enabled most cancer patients (approximately 60%) to live for a considerable period following diagnosis (46). Therefore, stigmatisation varies by stage and cancer continuum (treatment vs remission). “Disruptiveness” refers to how much cancer interferes with relationships and communication. Impaired psychosocial functioning or a poor prognosis among cancer survivors may impede contact with others who are hesitant or unsure how to approach them (15, 19). “Aesthetic” refers to the degree to which cancer makes people aesthetically unpleasant. Bilateral mastectomy or unsymmetrical breasts can cause significant disfigurement. Female chemotherapy-induced alopecia may be more aesthetically unpleasant than in male patients. Aesthetic concerns were mostly expressed by younger participants in this study. “Origin” refers to whether patients are thought to be responsible for their cancer (e.g., smoking, obesity, poor diet, or lifestyle). Because breast cancer is sometimes thought to be hereditary (due to genetic susceptibility (e.g., BRACA1/2 germline and family history)) (3), having a family member with cancer makes people feel as if their feeling of security is jeopardised or that they are not immune to cancer.

Associative or secondary stigma refers to the experience of stigma by relatives or friends of stigmatised group members or among healthcare practitioners who offer treatment to stigmatised group members (5). This was also identified among breast cancer survivors and is consistent with Arab culture. Arab women feared their husbands would divorce them or take a second wife if they were no longer fertile or sexually attractive, compromising family integrity (46). Protecting and promoting the well-being of their families was a top priority for many Jordanian and Arab women. Some mothers hide their diagnoses from their children to spare them sadness. Our findings suggest internalised stigma may undermine mothers’ protective sensations, especially in extended families (47, 48). In their sociocultural context, it is not uncommon for Arab women who are aware of a family history or genetic predisposition to assume that their daughters or sisters have a lower likelihood of marrying or having children and to blame themselves. As a result, people prefer not to share anything that could jeopardise or stigmatise their family’s integrity or reputation (47–49), and families frequently respond to social stigma and its attendant harm to family well-being by maintaining strict concealment regarding breast cancer (47–49). This can lead to delays in seeking medical evaluation or early detection (50). This is of concern considering that the median age of the study population is relatively young.

The second theme describes stigma resilience and empowerment based on sociocultural change and coping strategies used by survivors to counter the stigmatisation process. Our findings show that empowered breast cancer survivors may play a key role in promoting early detection by sharing positive experiences of successful breast cancer survivorship. Cultural awareness programmes should go beyond early diagnosis to shed light on the survivorship phase, improve public understanding of “survivorship” identity, debunk misconceptions, and provide specific psychological support initiatives to boost survivors’ health wellbeing. Awareness campaigns must be tailored to the different sociocultural norms and religious standards of the community or subcommunities, as well as the specific needs of survivors. It should reduce fear and stigma and encourage routine screenings. The goal of cancer education is to increase people’s knowledge of the causes and prognosis of breast cancer, addressing its progression, aetiology, disruptiveness, and risk (14). Further, the media is a key cultural venue that promotes illness-related structural stigma, such as in the workplace, so transformative reforms are needed in how cancer topics are communicated to the public. Self-compassion, positive acceptance of diagnosis, and self-disclosure of illness are partially aligned with earlier diagnosis and improve resilience (51). Islamic religion and religious fatalism were also good influences on resilience; this is congruent with research on Islamic philosophy of disease. In this respect, breast cancer was seen to be a test of faith delivered by God (47, 52). To pass this exam, survivors interpreted it as a call for tolerance, patience and acceptance. Such behaviour would be forever rewarded through the atonement of sins and reward in the hereafter (“*Al-Akhira”*).

### Study strengths and limitations

The study was conducted in one hospital. Despite this, Jordanian cancer survivors are typically treated in multiple treatment facilities due to resource constraints. As a result, their interactions with the entire healthcare system may be reflected in their experiences. limitations of the study were that it only included Jordanian cancer survivors who were eligible for government treatment funding schemes. Non-Jordanians with cancer were not included, even though some are refugees with greater unmet needs due to existing socioeconomic inequities. Moreover, the study included a homogeneous group of cancer survivors, most of whom were in stages I and II. Only two survivors with incurable metastatic disease underwent salvage chemotherapy for stages III and IV. For advanced incurable cancers, further research is warranted as the stigmatisation process varies with the course of disease progression (i.e. early vs. relapse or metastatic) cancer.

## Conclusions

Breast cancer-related stigma is a latent and hidden burden of the disease during survivorship, according to this study’s findings. Health policy makers and oncology practitioners need to allocate more attention and resources to reducing cancer-related stigma among the public and creating psychosocial supportive programmes for cancer survivors who are at risk of stigmatisation.

## Data availability statement

The original contributions presented in the study are included in the article/**Supplementary Material**. Further inquiries can be directed to the corresponding author.

## Ethics statement

This qualitative study was approved by Jordan University Hospital (JUH) approval number: (10/2019/8990) and Kingston’s university ethical requirements for scientific research (Approval number/1416). Before the study, participants gave written and verbal consent. Specifically, verbal consent was obtained from members participating in online group discussions. The patients/participants provided their written informed consent to participate in this study.

## Author contributions

Conceptualization: SJM, RK, and SN-G; Methodology: SJM, RK, and SN-G; Data collection and transcriptions: SJM; Formal analysis: SJM, RK, and SN-G; Writing and Editing: SJM and RK; Supervision and Revision: RK and SN-G. All authors contributed to the article and approved the submitted version.
