# Supplementary material for: Latency of breast cancer stigma during survivorship and its influencing factors: A qualitative study
Source: Front Oncol. 2023 Mar 14;13:1075298. doi: 10.3389/fonc.2023.1075298 (PMC10043425; doi:10.3389/fonc.2023.1075298)
Supplement: Supplementary file 1 [file DataSheet_1.docx]

**Appendix 1. Interviews and Focus groups schedules**

**Part 1: Interviews with Breast Cancer survivors**

| 1. **Firstly, I would like to talk about your *Journey with cancer* from time of diagnosis.**    1. Tell me about your journey with cancer, how it was diagnosed, how was the treatment process.   Useful probes: tell me more? Why? Explain to me? Can you give me an example? Give me a picture, a description of the whole process, how it goes?  1.1.2. Tell me about your experience (challenges difficulties, struggle) with cancer? (Individual level, family, job-related, society, ask about different stages, use probes)  1.1.3. Let’s talk about the treatment; tell me more about the counselling process? How you were counselled about the medications, treatment plans & treatment side effects?   - 1. **I’d like you to share your experiences on any obstacles during your treatment process? How were your concerns being dealt with?**       1. How did you feel about the adequacy & quality of information you received from the health teams? Were there any communication barriers? What have you done to overcome any barriers to access health services?   2. Did you have any particular concerns about the treatment side effects (Y/N)? Were your concerns fully addressed?   Prompts: how well did you understand the information supplied? Did you get any written information, like leaflets or informational booklets? what resources were needed and not supplied   - - 1. how did you deal with any treatment-related adverse events post-discharge?   1. **Let’s focus on the follow up treatment. Now you are visiting your doctor for a follow up, tell me about your typical journey? How does it usually go? if follow up is completed, how long did it take you until you finished your follow up?**      1. Can you describe the typical activities you do in the process? Which activities take the longest waiting times?   *Prompts*: did you know what to expect? Did you know the follow up plan, appointments, treatments, tests? What were the challenges of this period? What coping mechanisms or support strategies did you use? |
| --- |

| 2. **Communications with Healthcare professionals** |
| --- |
| - 1. **Focusing on communication, tell me how you feel about the communication with the medical team? Which form of communication was mostly used? For example, face to face, phone e-consultation, chatting, video consultation? Was communication consistent in quality and frequency throughout the treatment cycle and follow up?**       1. do you feel included in treatment plan? What were the most information needed/desired during those communications/consultations? Prompt: Can you tell me why?      2. Is there anything you feel can be improved or anything to be recommended that you would like to share? |
| 1. **Coping strategies** |
| - 1. **Focusing more on coping strategies, what are/were your coping/support strategies to combat the disease during or after completing your treatment?**   2. **How did you confronted the challenges (body image, emotional adjustment, spiritually, networking, role of religion, extent of changed identity, relationships with others?**   **3.2. What did others close to you do for you that made a difference?**  **3.3. Did you join a support group? If so, how did it help? If not, why?**  **3.4. Were there any online (apps, websites)/offline programs or services offered to you or you used as support or information during the treatment process? Were they helpful? What other support would you have liked**?  *Probes: Give me an example?*  *If online services were available/used, give me examples? Give me examples of alternative online resources you used to help you in your battle with cancer?*  *If any useful resource (like a website or mobile app) is available, would you use it?*  **4.Is there anything else you would like to share with us on the topic?** |

**Part 2: Focus groups interview schedules**

**This part aims to determine the potential features of such an application in light of your preferences, perspectives and experiences.**

**1.Tell me about your experience of using mobile applications? what type of apps do you use for personal and professional use? What about health/ fitness/ medication reminders/nutrition apps?**

**2.. In your opinion, what is the role and importance of mobile apps in supporting cancer patients and their informal caregivers?**

2.1. What are the benefits, advantages of such a digital solution as a supportive tool in your journey with cancer and the treatment plan?

**3. What would you like this app to offer**

3.1. What information would you like the app to have? (prompts: information about short and long side effects, how to manage between cycles, nutritional plans, effect of cancer on relationships, insurance, appointments, follow up schedules (mammogram, ultra-sound, colposcopy etc.)

3.2. What other features would you desire?

(prompts communication tool with HCP, side effect alert, medication reminder, online consultations? Communication with HCPs?).

3.3. Would you use it? What makes an app an attractive option to support your journey with cancer? At what stage of your journey with the disease would an app be most useful? (for example, during active treatment or after follow up).

**4. Focusing specifically on follow up, how can the app improve some aspects of the follow up?**

Prompts: ask about appointments, live tracking of the patient’s journey in the clinic, medication refill, waiting to get your medication ready? Medication shortage, how the app can help you to communicate with HCPs about such issues?

**5. With regards to emotional support, how can the application provide an emotional-social support to cancer patients?**

Prompts: ask about support groups, to what extent in Jordan people like to share their experiences via written and visual content (videos) or simply group meetings with peers? What are your thoughts on that? (prompts, why, explain, other opinions? etc)

What are the social and individual barriers that may facilitate/hinder using apps for social support?

**6. What about the disadvantages’ or pitfalls of a mobile app for cancer supportive care? Will you use it all the time or at certain times?**

prompts: can you explain why would you continue using it?

Prompts: what is the perceived value of different features? Do you think that all features will be useful during all phases of the disease? (Y/N)

Do you think that the mobile app can replace face to face consultation or enhance communications? (Y/N)

**7. Is there anything else you would like to share with us on the topic?**
